# Response: Commentary: A Novel Predictive Model to Estimate the Number of Mature Oocytes Required for Obtaining at Least One Euploid Blastocyst for Transfer in Couples Undergoing In Vitro Fertilization/Intracytoplasmic Sperm Injection: The ART Calculator

**DOI:** 10.3389/fendo.2020.598416

**Published:** 2020-11-27

**Authors:** Sandro C. Esteves, José F. Carvalho

**Affiliations:** ^1^ANDROFERT, Andrology and Human Reproduction Clinic, Campinas, Brazil; ^2^Statistika Consulting, Campinas, Brazil

**Keywords:** predictive model, age effect, mature oocytes, blastocyst transfer, euploidy, assisted reproductive technology

We thank Fischer and Baukloh for their commentary (1) on the subject matter of our article concerning a novel predictive model (ART Calculator) to estimate the minimum number of metaphase II (MII) oocytes required to obtain at least one euploid blastocyst for transfer in couples undergoing assisted reproductive technology (ART) (2). We developed the ART calculator to help clinicians objectively estimate the POSEIDON’s group metric of success in ART (3, 4). With the ART calculator, the POSEIDON’s metric can be estimated without preimplantation genetic testing for aneuploidy (PGT-A).

The model provides two types of predictions. Pre-treatment, it estimates the minimum number of MII oocytes to achieve ≥1 euploid blastocyst for transfer, and post-treatment, it provides a revised estimate of the probability of achieving ≥1 euploid blastocyst when fewer than the predicted number of MII oocytes are obtained (1). In practical terms, the ART calculator’s main output is the average minimum number of MII oocytes required for at least one euploid blastocyst, which increases progressively with the female age and is magnified further by the use of testicular sperm from patients with nonobstructive azoospermia. Accordingly, patient-oriented strategies to achieve the number of oocytes needed to obtain one euploid embryo for transfer may be elaborated to potentially increase success prospects (5, 6).

Following its publication, the ART calculator was validated in a multicenter study involving approximately 1,500 infertile patients subjected to IVF-ICSI and PGT-A (7). The validation study showed that the estimations provided by the ART calculator were strongly correlated with the actual probability of blastocyst euploidy per MII oocyte (r = 0.91) and the minimum number of MII oocytes required to obtain at least one euploid blastocyst (r = 0.88). In both the original and validation studies, female age was the primary factor affecting blastocyst euploidy among infertile couples undergoing ART (2, 7).

Fischer and Baukloh, in their analysis of our study, raised two valid points that warrant further elaboration. First, given the overall concordance of trophectoderm biopsy and polar body biopsy, the ART calculator’s estimation might be somewhat extrapolated for determining the number of MII oocytes needed to have at least one euploid MII oocyte; an intriguing hypothesis that warrants further evaluation.

Secondly, and more importantly, the authors discuss embryo ploidy’s intraindividual variation, which has relevance for patient counseling and treatment. Indeed, our studies show that there is variation in the probability of blastocyst euploidy within same-age women (2, 7, 8). To account for this variability, the ART calculator provides—in addition to the average minimum number of MII oocytes required for at least one euploid blastocyst—the 95% confidence interval of each estimation. Due to the intrinsic uncertainties in estimations of biological phenomena like embryo ploidy status, the ART calculator also allows users to set the desired probability of success (e.g., 70, 80, 90%) for predicting the number of MII oocytes. For example, according to the ART calculator applying a user-defined 80% probability of success, a patient of 39 years-old undergoing IVF-ICSI with ejaculated sperm will require 16 MII oocytes (95% CI 13–20) to obtain at least one euploid blastocyst for transfer (www.grouposeidon.com). This computation means that the predicted number of MII oocytes has an 80% chance of achieving at least one euploid blastocyst.

Along these lines, a novel finding from our research relates to the hypothesis of statistical independence of embryos concerning ploidy status. It implies that the ploidy status of a given embryo does not affect the probability of another embryo from the same cohort being euploid or aneuploid. In our validation study’s dataset, the negative binomial distribution fits the number of euploid blastocyst optimally (goodness-of-fit test: Pearson Chi-square = 4.577; Prob>Chi-square = 0.59; **Supplementary Figure (7)**). In our case, since the P < X2 = 0.59, the null hypothesis—the data come from a binomial distribution—is accepted. This type of distribution is consistent with the hypothesis of statistical independence. Therefore, we suggest that the negative binomial distribution is used for modeling in studies applying logistic regression analysis for assessing the effect of predictors on blastocyst ploidy status.

Lastly, Fischer and Baukloh shared their oocyte polar body biopsy and NGS analysis data, which indicate little difference in the number of euploid oocytes among women who had two consecutive PGT-A treatments. We also looked at whether these findings hold for blastocysts. For this, we examined our dataset of 747 consecutive patients subjected to IVF-ICSI with ejaculated sperm and PGT-A by NGS over a two-year period. Logistic regression analysis for the binary response ‘euploid blastocyst = yes/no’, accounting for age, cycle number, sperm source, paternal age, infertility factor, maternal BMI, and ovarian reserve markers (AFC and AMH) revealed that only female age (p <0.0001) is a relevant predictor of blastocyst euploidy. The probability of blastocyst euploid per MII oocyte progressively decreased with the female age, but it was not affected by whether the patient was on the first or second PGT-A cycle (L-R Chi-square 1.307; p = 0.52) (**Figure 1**). A total of 88 patients had two PGT-A cycles within a 6-month period. In this cohort, the PGT-A cycle number did not materially affect euploidy rates and the number of euploid blastocysts (mean ± SE): 34.9% and 0.77 ± 0.05 (cycle 1), and 36.2% and 0.69 ± 0.10 (cycle 2). Our data support the notion that the probability of an MII oocyte becomes a euploid blastocyst is reasonably constant across women, depending only on explanatory variables (predictors) that might affect the response. Moreover, our findings indicate that blastocyst euploidy rates are relatively stable across cycles of the same women within a short time frame.

**Figure 1:**
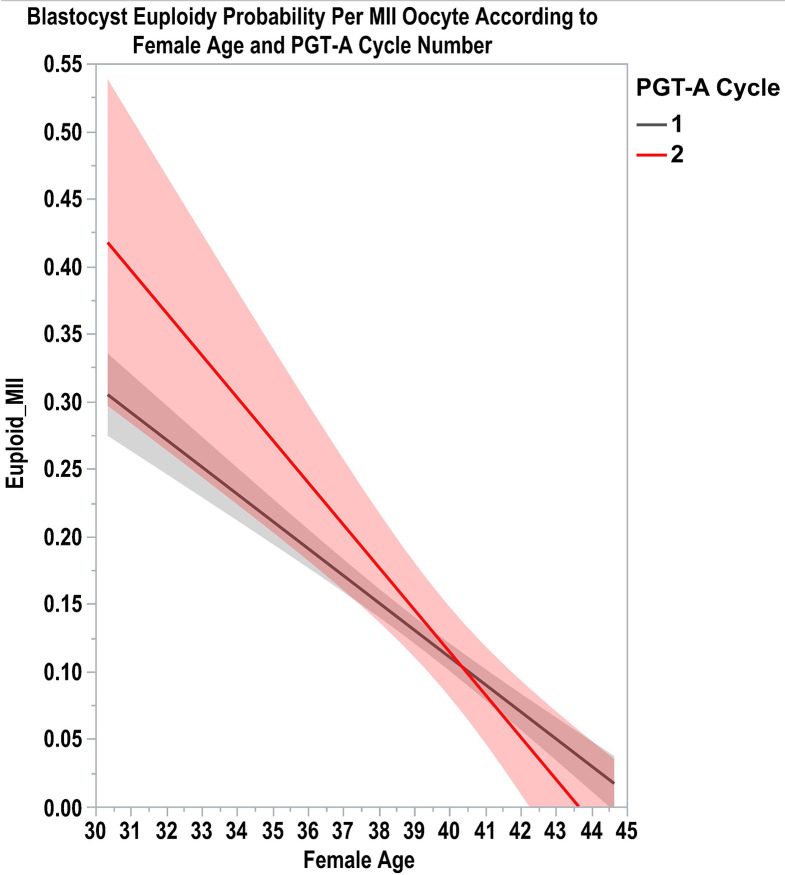
Lines of fit show a linear regression with 95% confidence intervals for continuous X (female age) and Y (euploid blastocyst per MII oocyte) probability according to PGT-A cycle number.

## Author Contributions

SE collected the data and drafted the commentary. JC analyzed the data and drafted the commentary. All authors contributed to the article and approved the submitted version.

## Supplementary Material

The Supplementary Material for this article can be found online at: https://www.frontiersin.org/articles/10.3389/fendo.2020.598416/full#supplementary-material

## Conflict of Interest

SE declares receipt of unrestricted research grants from Merck, and lecture fees from Merck. The funder listed above had no involvement with the ART calculator development. JC is co-owner of Statistika Consulting.
