# Supplementary figures and images for: Response: Commentary: A Novel Predictive Model to Estimate the Number of Mature Oocytes Required for Obtaining at Least One Euploid Blastocyst for Transfer in Couples Undergoing In Vitro Fertilization/Intracytoplasmic Sperm Injection: The ART Calculator
Source: Front Endocrinol (Lausanne). 2020 Nov 27;11:598416. doi: 10.3389/fendo.2020.598416 (PMC7731887; doi:10.3389/fendo.2020.598416)

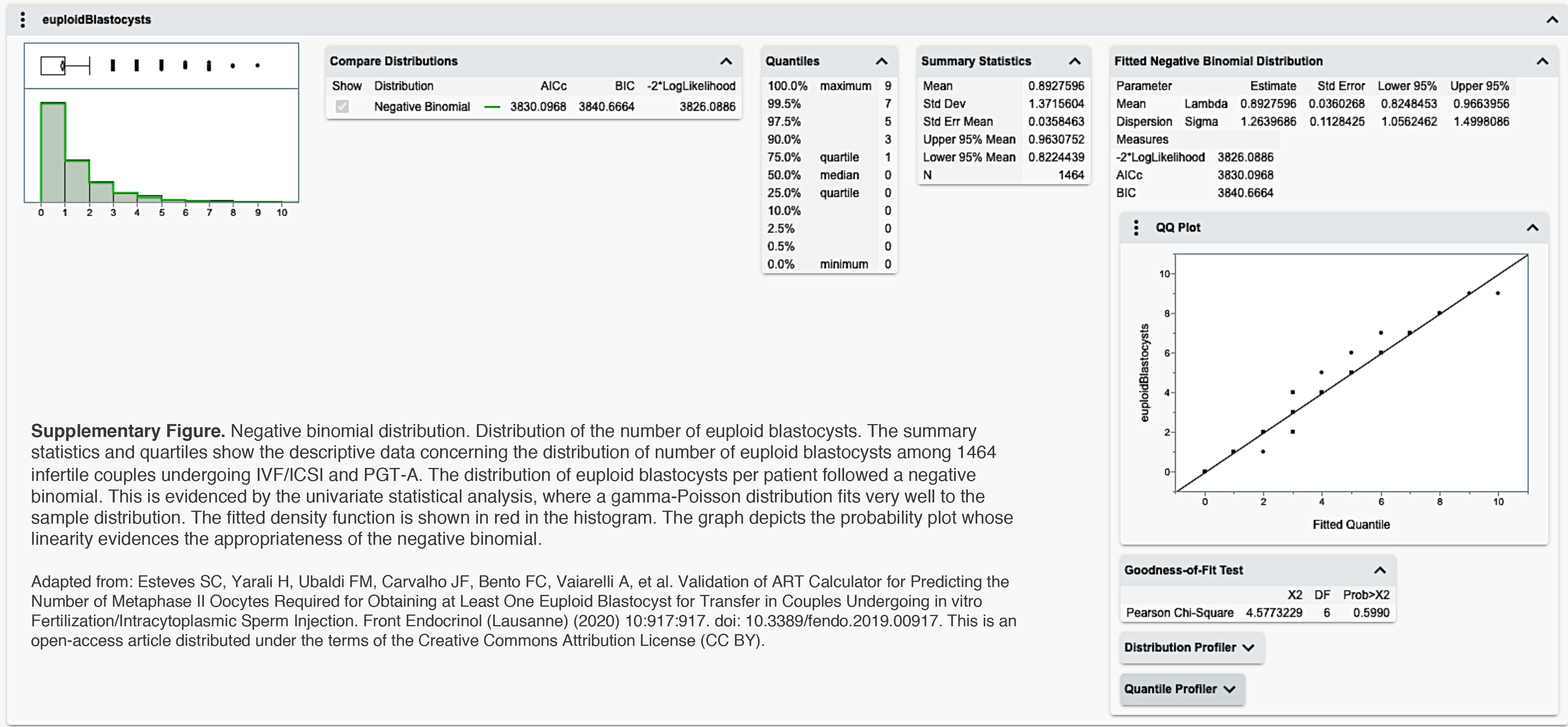

Supplement: Supplementary file 1 [file Image_1.tif]
